# Metastatic Uveal Melanoma: Treatment Strategies and Survival—Results from the Dutch Melanoma Treatment Registry

**DOI:** 10.3390/cancers11071007

**Published:** 2019-07-18

**Authors:** Anouk Jochems, Monique K. van der Kooij, Marta Fiocco, Maartje G. Schouwenburg, Maureen J. Aarts, Alexander C. van Akkooi, Franchette W.P.J. van den Berkmortel, Christian U. Blank, Alfonsus J.M. van den Eertwegh, Margreet G. Franken, JanWillem B. de Groot, John B.A.G. Haanen, Geke A.P. Hospers, Rutger H. Koornstra, Wim H.J. Kruit, Marieke Louwman, Djura Piersma, Rozemarijn S. van Rijn, Karijn P.M. Suijkerbuijk, Albert J. ten Tije, Gerard Vreugdenhil, Michel W.J.M. Wouters, Michiel C.T. van Zeijl, Koos J.M. van der Hoeven, Ellen Kapiteijn

**Affiliations:** 1 Department of Medical Oncology, Leiden University Medical Centre, Albinusdreef 2, po box 9600, 2300 RC Leiden, The Netherlands; 2 Dutch Institute for Clinical Auditing, Rijnsburgerweg 10, 2333 AA Leiden, The Netherlands; 3 Medical Statistics, Department of Biomedical Data Sciences, Leiden University Medical Centre, Albinusdreef 2, po box 9600, 2300 RC Leiden, The Netherlands; 4 Mathematical Institute Leiden University, Niels Bohrweg 1, 2333 CA Leiden, The Netherlands; 5 Department of Medical Oncology, Maastricht University Medical Centre, P. Debyelaan 25, 6202 AZ Maastricht, The Netherlands; 6 Department of Surgical Oncology, Netherlands Cancer Institute—Antoni van Leeuwenhoek Hospital, Plesmanlaan 121, 1066 CX Amsterdam, The Netherlands; 7 Department of Medical Oncology, Zuyderland Medical Centre, 6130 MB Sittard-Geleen, The Netherlands; 8 Department of Medical Oncology, Netherlands Cancer Institute—Antoni van Leeuwenhoek Hospital, Plesmanlaan 121, 1066 CX Amsterdam, The Netherlands; 9 Department of Medical Oncology, VU Medical Centre, de Boelelaan 1117, 1081 HZ Amsterdam, The Netherlands; 10 Department of Health Technology Assessment, Institute for Medical Technology Assessment and Erasmus School of Health Policy & Management, Erasmus University, Burgemeester Oudlaan 50, 3000 DR Rotterdam, The Netherlands; 11 Department of Medical Oncology, Isala Oncological Center, 8000 GK Zwolle, The Netherlands; 12 Department of Medical Oncology, University Medical Centre Groningen, Hanzeplein 1, 9713 GZ Groningen, The Netherlands; 13 Department of Medical Oncology, Radboud University Medical Centre, Geert Grooteplein Zuid 10, 6500 HB Nijmegen, The Netherlands; 14 Department of Medical Oncology, Erasmus MC Cancer Institute, Molewaterplein 40, 3000 CA Rotterdam, The Netherlands; 15 Netherlands Comprehensive Cancer Organisation, PO Box 19079, 3501 DB Utrecht, The Netherlands; 16 Department of Medical Oncology, Medisch Spectrum Twente, Koningsplein 1, 7512 KZ Enschede, The Netherlands; 17 Department of Medical Oncology, Medical Centre Leeuwarden, Henri Dunantweg 2, 8934 AD Leeuwarden, The Netherlands; 18 Department of Medical Oncology, University Medical Centre Utrecht, Heidelberglaan 100, 3584 CX Utrecht, The Netherlands; 19 Department of Medical Oncology, Amphia Ziekenhuis, Langendijk 175, 4819 EV Breda, The Netherlands; 20 Department of Medical Oncology, Maxima Medisch Centrum, de Run 4600, 5500 MB Veldhoven, The Netherlands

**Keywords:** uveal melanoma, metastatic uveal melanoma, survival, treatment strategy, prognostic factor

## Abstract

Uveal melanoma (UM) is the most common primary intraocular tumor in adults. Up to 50% of UM patients will develop metastases. We present data of 175 metastatic UM patients diagnosed in the Netherlands between July 2012 and March 2018. In our cohort, elevated lactate dehydrogenase level (LDH) is an important factor associated with poorer survival (Hazard Ratio (HR) 9.0, 95% Confidence Interval (CI) 5.63–14.35), and the presence of liver metastases is negatively associated with survival (HR 2.09, 95%CI 1.07–4.08). We used data from the nation-wide Dutch Melanoma Treatment Registry (DMTR) providing a complete overview of the location of metastases at time of stage IV disease. In 154 (88%) patients, the liver was affected, and only 3 patients were reported to have brain metastases. In 63 (36%) patients, mutation analysis was performed, showing a GNA11 mutation in 28.6% and a GNAQ mutation in 49.2% of the analyzed patients. In the absence of standard care of treatment options, metastatic UM patients are often directed to clinical trials. Patients participating in clinical trials are often subject to selection and usually do not represent the entire metastatic UM population. By using our nation-wide cohort, we are able to describe real-life treatment choices made in metastatic UM patients and 1-year survival rates in selected groups of patients.

## 1. Introduction

Uveal melanoma (UM) is the most common primary intraocular tumor in adults and arises from the melanocytes residing in the stroma [1,2]. Between 2012 and 2018, the incidence of primary uveal melanoma was approximately 200 new cases per year in the Netherlands [3]. European data on the incidence of primary uveal melanoma report 4.4 cases per million in Europe [4]. Among all intraocular melanomas, choroidal melanomas occur most frequently (80–90% of cases), but tumors may also develop in the iris or ciliary body [2]. The diagnosis of uveal melanoma is based on non-invasive testing techniques, such as fundoscopy or ultrasound, performed by an experienced clinician. Ocular treatment of uveal melanoma consists of enucleation (“radical treatment”) or radiotherapy, usually in the form of plaque brachytherapy or proton radiotherapy (“conservative treatment”) [5]. Management of primary uveal melanoma is guided by the size and location of the tumor, presence of extraocular extension, visual potential and patient age and preference. In selected patients, both treatment modalities show similar survival and risk of metastases, with radiotherapy having the advantage of a better cosmetic result and the possibility of saving vision in the smaller tumors [6].

Unfortunately, up to 50% of patients with uveal melanoma will ultimately develop metastatic disease. The most frequently affected metastatic site is the liver [4,6,7]. The site of the metastases has an impact on survival; patients with liver metastasis have a poorer prognosis than patients with extrahepatic metastasis [8,9]. Previously, it was thought that there would be no survival advantage in early diagnosis of metastatic disease because of the lack of standard-of-care therapy for metastatic uveal melanoma. However, patients with early diagnosis of metastatic disease might benefit from liver-directed therapy, which is associated with clinical utility [10,11,12,13,14,15] or they might benefit from participation in a clinical trial. Under the Dutch and UK uveal melanoma guidelines [16,17], patients with primary uveal melanoma are therefore advised to have 6-monthly liver function tests in combination with liver-specific imaging by a non-ionizing modality to detect metastatic disease in an earlier phase.

On a molecular level, uveal melanomas differ significantly from cutaneous melanomas. Unlike cutaneous melanoma, uveal melanoma is not characterised by frequent BRAF or NRAS mutations, so that advances in targeted therapy for cutaneous melanoma are not applicable to metastatic uveal melanoma. Early activating mutations in GNAQ or GNA11 are present in about 80% of primary uveal melanomas. These lead to activation of downstream signalling pathways. [18] Inactivating somatic mutations are present in the gene encoding BRCA1-associated protein 1 (BAP1) in more than 80% of metastasising tumors, implicating a role in the progression of uveal melanoma. [19] Mutations in SF3B1 and EIF1AX in primary uveal melanoma are associated with a relatively good prognosis. [20,21] Greater understanding of the molecular pathogenesis may provide opportunities for patients who benefit from surveillance and may eventually provide specific targeted therapy for metastatic uveal melanoma patients.

Over the past few years, different treatment strategies have been evaluated in patients with metastatic uveal melanoma. The best responses have been reported with local treatment strategies in patients with exclusive and limited hepatic metastasis in whom surgical resection, isolated hepatic perfusion with melphalan, radiotherapy, radiofrequency ablation or radio-embolization was performed [10,11,12,13,14,15]. In patients with diffuse liver metastases or extensive extrahepatic metastases, systemic therapy is the only treatment strategy available. Several combinations of drugs have been investigated in phase Ib/II/III trials in patients with metastatic uveal melanoma. Until now, none of the systemic treatments with chemotherapy, [22,23,24] immune checkpoint inhibitors [25,26,27,28,29,30,31,32] or targeted therapy, [33,34] have shown substantial efficacy in metastatic uveal melanoma.

In this article, we present data from our Dutch cohort of metastatic uveal melanoma patients describing affected metastatic sites, mutation analysis, clinical characteristics associated with survival and treatment choices made and the corresponding one-year survival. By describing these groups of patients, we show the impact of clinical characteristics and selecting metastatic UM patients for treatment in our real-life population.

## 2. Results

### 2.1. Patient Characteristics

**Figure 1:**
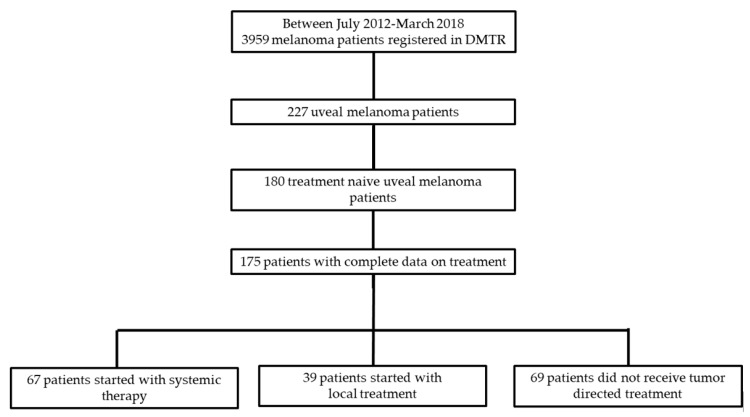
Nation-wide cohort of metastatic uveal melanoma patients registered in the Dutch Melanoma Treatment Registry (DMTR): All patients with complete data on treatment were analyzed and subdivided based on the first treatment option when diagnosed with metastatic disease.

Of the 3959 registered patients in the DMTR, a total of 175 metastatic uveal melanoma patients were identified for analysis (Figure 1). Baseline characteristics are presented in Table 1.

**Figure 2:**
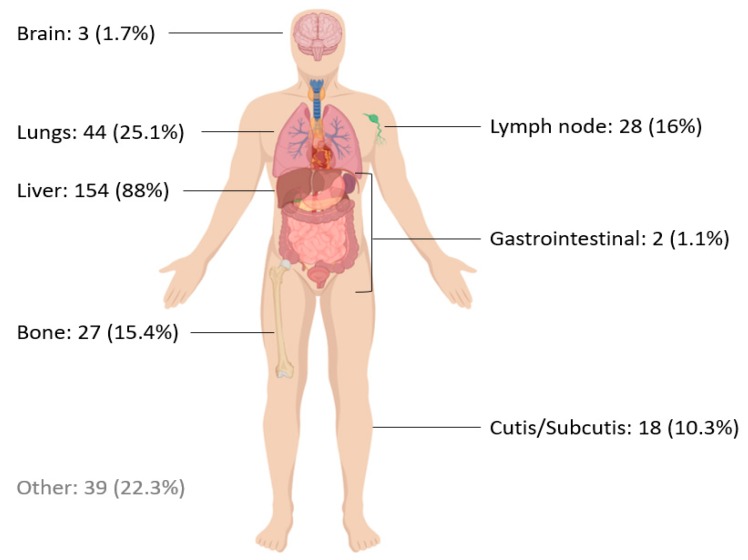
Frequency of affected organ in our cohort of patients with metastatic uveal melanoma. (More than one organ can be affected).

The median age of metastatic UM patients in this cohort was 65 years. The majority of patients (74.9%) scored well on the World Health Organization (WHO) performance scale (0–1). Lactate dehydrogenase level (LDH) was elevated in 85 (48.6%) patients (Table 1). The liver was the most affected site: 88% of patients having liver metastases. Other affected sites were the lungs (25.1%), lymph nodes (16%) and bones (15.4%) (Figure 2). Differences in clinical characteristics between the treatment groups are presented in Table 1.

### 2.2. Mutation Analysis

**Figure 3:**
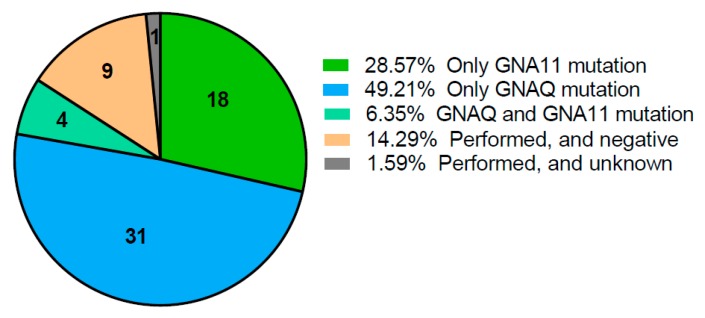
Results of molecular analysis of GNAQ/GNA11 mutation. Analysis was performed in 63 of 175 patients (36%).

Molecular analysis of the activating mutation in the GNAQ or GNA11 genes was performed in 63 patients (36%) (Figure 3). The fact that detection of these mutation was of no therapeutic consequence might explain why these genes were not included in a standard NGS panel. In 31 of these 63 (49.2%) patients a mutation in the GNAQ was discovered and in 18 patients (28.6%) a GNA11 mutation was confirmed. These results are consistent with the known literature describing most primary uveal melanoma having a GNAQ or GNA11 mutation [18,35].

### 2.3. Treatment of Metastatic UM Patients

**Figure 4:**
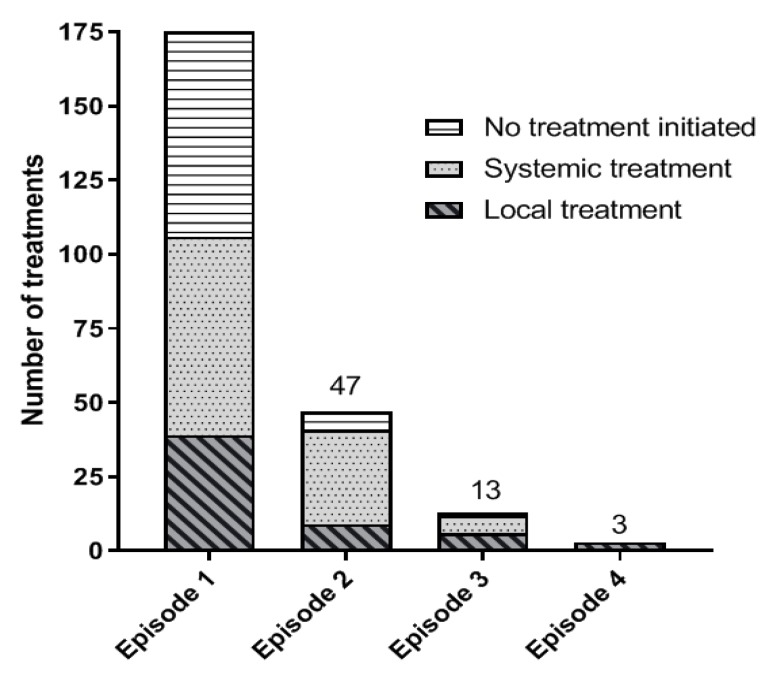
Treatment strategies per treatment episode. Some patients received more than one line of treatment after failure of first-line treatment. (treatment episode 1: treatment strategy performed when diagnosed with metastatic uveal melanoma, treatment episode 2: second treatment strategy after failure of first-line treatment etc.).

In our study, 67 patients (38.3%) received systemic therapy when diagnosed with metastatic disease. Several systemic drug regimens were applied, both in- and outside a clinical trial setting as there is no standard of care for patients with metastatic uveal melanoma. These regimens consisted of chemotherapy with dacarbazine, immune checkpoint inhibitors or targeted drugs. Several different clinical trials, varying from phase I to phase III trials, were open for patient enrollment at different time windows in the investigated period. All patients receiving a targeted drug participated in a clinical trial; for example, in the NCT01430416 trial (phase 1 trial with AEB071), NCT01801358 trial (phase 1b/II study with AEB071 + MEK162), NCT01974752 trial (phase 3 trial with selumetinib, or NCT02601378 (phase 1 trial with LXS196). In addition, patients could be included into the N11RFA trial, a phase II study exploring the combination of ipilimumab with RFA. Fifty-three (79.1%) of 67 patients were treated in a clinical trial as a part of first-line systemic therapy. Some patients received more than one treatment after the failure of first-line therapy. During registration, a total of 108 systemic therapies were given, in total 85 (78.7%) of these treatments were part of participation in a clinical trial (Figure 4).

**Figure 5:**
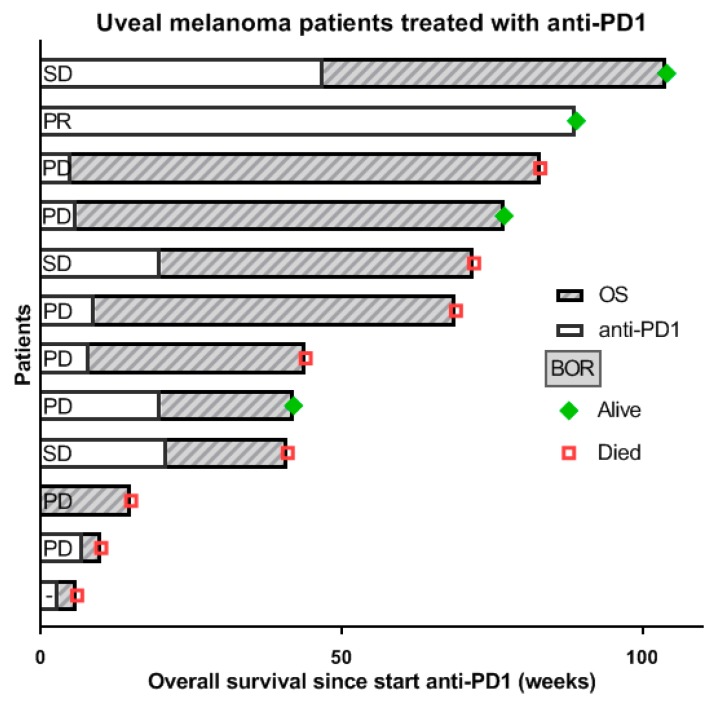
Best response and survival of 12 metastatic UM patients treated with an anti-PD1 antibody (no clinical trial participation).

Sixteen patients received systemic treatment with a checkpoint inhibitor outside a clinical trial setting. Four patients received the anti-CTLA-4 antibody ipilimumab and 12 patients received an anti-PD1 antibody One patient was treated with the combination of anti-CTLA-4 and anti-PD1 therapy. As most patients treated with anti-CTLA-4 antibody were included in a clinical trial (as part of a phase II study exploring the combination of ipilimumab with RFA, EudraCT Number: 2011-004200-38), overall survival data for this group are not yet available. The median OS of these 12 patients treated with an anti-PD1 antibody was 54.3 weeks, ranging between 6 and 104 weeks. Data on duration of treatment, best overall response and overall survival are shown in Figure 5. Median follow-up computed with reverse Kaplan- Meier was equal to 89 weeks (95% CI 70.76–107.24).

Thirty-nine patients (22.3%) received local treatment when first diagnosed with metastatic uveal melanoma. These local treatment regimens included surgical resection of metastases, isolated hepatic perfusion with melphalan, radiotherapy, radiofrequency ablation or radio-embolization. Sixty-nine patients (39.4%) did not receive anti-tumor directed therapy but received best supportive care (Figure 1).

### 2.4. Survival

**Figure 6:**
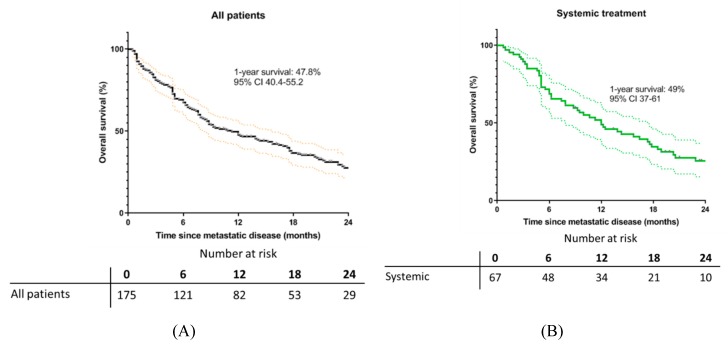

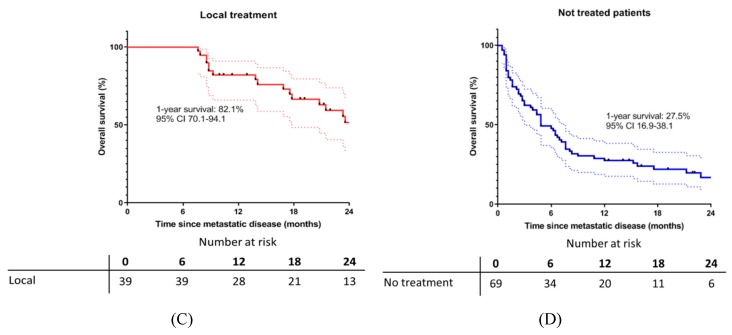
Kaplan-Meier Estimates for all 175 metastatic UM patients and per treatment strategy administered when diagnosed with metastatic disease. (**A**) Kaplan-Meier (KM) estimate for all metastatic UM patients, (**B**) KM estimate for patients treated with systemic therapy, (**C**) KM estimate for patients with local treatment, (**D**) KM estimate for patients receiving no tumor directed treatment (best supportive care.

The median follow-up was computed with reverse Kaplan-Meier (where the event indicator is reversed so that the outcome of interest is censored [36] and was equal to 120 weeks (95% CI 96.3–143.7). One year after the diagnosis of metastatic uveal melanoma, 47.8% of all patients were alive (95% CI 40.4–55.2). There is a considerable difference in survival at one year among patients belonging to different treatment groups and patients included in the BSC-group. The prognosis at one-year observed in patients receiving systemic therapy or local therapy was 49% (95% CI 37–61) and 82.1% (95% CI 70.1–94.1), respectively. One-year survival for patients receiving best supportive care was equal to 27.5% (95% CI 16.9–38.1) (Figure 6).

**Figure 7:**
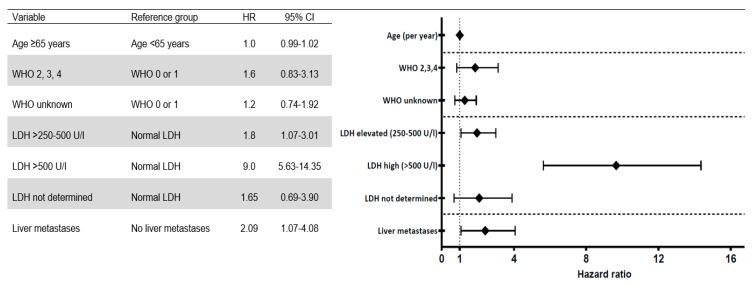
Multivariable hazard ratios (HR) associated with poorer survival in the full cohort along with the 95% confidence interval (CI).

The multivariable Cox analysis showed that slight to moderately elevated LDH (250–500 U/L) and high LDH level (>500 U/L) were a statistically significant factor associated with poor survival (*p* < 0.001), HR of 1.8 (95% CI 1.07–3.01) and 9.0 (95% CI 5.63–14.35) respectively. Also, the presence of liver metastases was negatively associated with survival, HR 2.09 (95% CI 1.07–4.08, *p* = 0.03). A WHO performance score >1 on its own seemed to be associated with poorer survival in a univariable Cox analysis. However, when included in the multivariable analysis this association was no longer statistically significant. “Age” as a continuous variable was included in the model, but was not statistically significant (HR 1.0 (95% CI 0.99–1.02), *p* = 0.69). (Figure 7).

Figure S1 shows Kaplan-Meier estimates for survival when patients are categorized according to non-elevated versus elevated serum LDH for all three treatment groups at baseline. Both in the group of patients not receiving tumor-directed treatment (BSC) and the systemically treated group, an LDH above 250 U/l was clearly associated with poorer survival (*p* < 0.001). However, in the local treatment group, this difference was not statistically significant (*p* = 0.15).

## 3. Discussion

Metastatic uveal melanoma has a poor prognosis, usually leading to rapid clinical decline and early death. According to the literature, the majority of patients survive for less than 12 months [7,8]. In our cohort, we analysed 175 patients with metastatic uveal melanoma according to first-line treatment strategies administered when they were diagnosed with stage IV disease between July 2012 and March 2018. The real-world results of this observational cohort are a reflection of uveal melanoma care available in the Netherlands and this article does not compare different treatment strategies and/or the impact on patient outcome. In our cohort, one-year survival for all patients with metastatic uveal melanoma is equal to 47.8% (95% CI 40.4–55.2), similar to that reported in known publications [7,8]. Studies reporting on survival in metastatic uveal melanoma have found the best results in terms of survival among patients in whom surgery or ablative procedures can be performed and among patients with solitary hepatic metastases [10,11,12,13,14,15]. Overall, these findings are suggestive of survival benefit, although it is likely that there is a selection bias towards the most clinically fit patients [9]. Based on the results in literature, the first choice of treatment in the Netherlands is, whenever possible, surgery, ablative procedures or isolated hepatic perfusion with melphalan (in a clinical trial setting). In line with the literature, our cohort shows a selection of relatively younger patients, with good WHO performance score, fewer metastatic sites and less elevated LDH who were treated with local treatment options. As no systemic therapy has been shown to improve overall survival for patients with metastatic uveal melanoma, there is no specific standard of care and patients should be directed to clinical trials. In the Netherlands, metastatic melanoma care has been centralised to 14 expert centers [36] improving management of metastatic melanoma patients, but also facilitating enrollment in clinical trials to get evidence-based treatment protocols. In our cohort in total 85 systemic therapies were given in the context of a clinical trial, to 63 unique patients. The lack of availability of clinical trials was sometimes a reason to provide systemic therapy outside a clinical trial setting. These systemic therapies were registered for treatment of metastatic cutaneous melanoma and given to patients with metastatic uveal melanoma. In the present situation, decision making on available treatment options in metastatic UM patients occurs mainly on clinical characteristics leading to selection of patients for treatment in- and outside a clinical trial. The limited efficacy of checkpoint inhibitors in uveal melanoma has led to the agreement among members of the Dutch Working Group on immunotherapy and oncology (WIN-O) not to treat patients with immune checkpoint inhibitors outside a clinical trial. Combination studies on ipilimumab/nivolumab and novel immune-based approaches might be more promising [37].

In our cohort of UM patients, classic risk factors associated with survival, as elevated LDH and the presence of liver metastases [7,8] are confirmed to be negatively associated with survival (Figure 7). The distribution of metastases (Figure 2) in our cohort is consistent with data from the large Collaborative Ocular Melanoma Study trials [38].

Our observational cohort may suffer from limitations in terms of the registration of real-world data, sometimes leading to missing variables which might affect results, especially in smaller treatment groups. For instance, in the group of patients receiving local treatment (39 patients) information on WHO performance score was missing in 14 patients (35.9%). Another registration flaw was detected in the documentation of the molecular analysis, reporting a GNAQ and GNA11 mutation in 6.4% of the analysed patients. These mutations are mutually exclusive. Other limitations relate to the choice of data to collect in a registry. From a scientific perspective, a broad set of clinical and pathological characteristics (including molecular and genomic alterations), treatment strategies, adverse events and survival is desirable. This is, however, not always feasible, and ongoing developments are more difficult to incorporate. At this time, the DMTR contains limited data on molecular and genomic tumor alterations.

Important strengths of our observational cohort are the complete overview of patient and metastatic tumor characteristics and treatment options available in the Netherlands between 2012 and 2018 for metastatic uveal melanoma patients. Differences in metastatic UM patients are most probably caused by differences in baseline characteristics and patient selection for specific treatment. However, this overview might be used by other authors for comparing survival between treatment groups and the impact of their treatment strategy applied.

## 4. Patients and Methods

### 4.1. Datasource

Since 2013, all Dutch metastatic melanoma patients have been referred to one of the 14 melanoma expert centers in the Netherlands. This centralisation of metastatic melanoma patients and the registration in the Dutch Melanoma Treatment Registry (DMTR), (providing nation-wide coverage retrospectively starting from July 2012), was initiated to assure safety and quality of melanoma care in the Netherlands [36]. Since the DMTR was set up, all patients with metastatic melanoma have been included in the registry, irrespective of the type of primary melanoma (i.e., cutaneous, uveal, or mucosal melanoma). The DMTR provides aggregated data information on basic patient and tumor characteristics, treatment regimens, grade 3 and 4 treatment related adverse events (according to the Common Terminology Criteria for Adverse Events, version 4.0) and clinical outcomes.

In compliance with Dutch regulations, the DMTR was approved by a medical ethical committee (METC Leiden University Medical Centre, 3 September 2013) and is not considered subject to the Medical Research Involving Human Subjects Act. All data are collected anonymously and only aggregated data are available for research and quality improvements. Data extraction from medical files is performed by data-employees. No informed consent will be signed, but patients are offered an opt-out possibility if they do not want their data registered in the DMTR. For this study, the data cut-off date was 25th March 2018.

### 4.2. Patients

Between July 2012 and March 2018, 227 patients with metastatic uveal melanoma were registered in the DMTR. Patients who received treatment before the DMTR was set up were excluded from analysis (Figure 1). We analysed 175 treatment-naive patients according to the type of treatment initiated at first presentation with metastatic disease: i.e., patients could be receiving: (i) systemic therapy, (ii) local treatment, or (iii) no tumor-directed therapy, but best supportive care (BSC). For this manuscript, we analysed only patients who had their first treatment post July 2012.

Systemic therapy included a variety of regimens with chemotherapy, immune checkpoint inhibitors and targeted drugs. Local treatment strategies included surgical resection, isolated hepatic perfusion with melphalan, radiotherapy, radiofrequency ablation or immune-embolization. Treatment strategies were performed either as standard care or in the context of participation in a clinical trial.

### 4.3. Statistical Analysis

Descriptive statistics were employed to summarise patient baseline characteristics on registration in the DMTR. To test the difference between categorical variables for different treatment strategy groups, a chi-square test was applied (Table 1). A rank-sum test has been used to test the difference between the median time from diagnosis to stage IV disease between groups of patients. Survival from the diagnosis of metastatic disease, was estimated according to Kaplan-Meier’s method. Median follow up was computed with reverse Kaplan-Meier method [39].

A univariable Cox analysis using variables “age” (age as a continuous variable), “gender” (male versus female), “WHO performance score” (WHO 0–1 vs. WHO ≥ 2), “LDH level” (elevated vs. non-elevated LDH) and the “presence of liver metastases” was performed. Subsequently, a multivariable Cox regression model was estimated, including the variables known to influence survival in metastatic cutaneous melanoma patients. All statistical analyses were conducted using SPSS (SPSS, version 23, IBM Corp. released 2015, Armonk, NY, USA).

## 5. Conclusions

We present baseline characteristics, mutation analysis and treatment strategies with the corresponding one-year survival of a nation-wide (full coverage) cohort of 175 patients with metastatic uveal melanoma in the Netherlands. Selection of patients for treatment was mainly based on clinical characteristics, showing elevated LDH (HR 9.0, 95% CI 5.63–14.35), and the presence of liver metastases (HR 2.09, 95% CI. 1.07–4.08) was negatively associated with survival in metastatic UM. The analysis of our observational cohort reflects the treatment choices made by physicians in Dutch melanoma expert centers. Our overview might be used by other authors for comparing survival between treatment groups and the impact of treatment strategy applied.

## Figures and Tables

**Table 1:** Patient characteristics at the moment of diagnosis with metastatic disease.

Patient Characteristics	All Patients (*n* = 175)	Systemic Therapy (*n* = 67)	Local Therapy (*n* = 39)	Best Supportive Care (*n* = 69)	*p*-Value
Age					0.001
Median, years (range)	65 (29–89)	61 (29–80)	61 (41–80)	69 (45–89)	
<65 years (%)	86 (49.1)	39 (58.2)	25 (64.1)	22 (31.9)	
>65 years (%)	89 (50.9)	28 (41.8)	14 (35.9)	47 (68.1)	
Gender (%)					0.98
Male	88 (50.3)	34 (50.7)	20 (51.3)	34 (49.3)	
Female	87 (49.7)	33 (49.3)	19 (48.7)	35 (50.7)	
WHO performance score (%)					0.000
0	106 (60.6)	55 (82.1)	21 (53.8)	30 (60.6)	
1	25 (14.3)	7 (10.4)	3 (7.7)	15 (21.7)	
2	11 (6.3)	2 (3)	1 (2.6)	8 (11.6)	
3	3 (1.7)	0	0	3 (4.3)	
4	1 (0.6)	0	0	1 (1.4)	
Unknown	29 (16.6)	3 (4,5)	14 (35,9)	12 (17.4)	
Median time from diagnosis primary tumor to stage IV					0.02 *
months (range)	38 (0–477)	43 (0–296)	29 (0–477)	42 (0–361)	
Brain metastases (%)					0.75
No	169 (96.6)	64 (95.5)	39 (100)	66 (95.7)	
Yes	3 (1.7)	2 (3)	0	1 (1.4)	
Unknown	3 (1.7)	1 (1.5)	0	2 (2.9)	
Liver metastases (%)					0.10
No	20 (11.4)	11 (16.4)	1 (2.6)	8 (11.6)	
Yes	154 (88)	56 (83.6)	38 (97.4)	60 (87)	
Unknown	1 (0.6)	0	0	1 (1.4)	
Metastatic sites (%)					0.002
<3 metastatic sites	134 (76.6)	44 (65.7)	39 (100)	52 (75.4)	
>3 metastatic sites	31 (17.7)	18 (26.9)	0	13 (18.8)	
Unknown	10 (5.7)	5 (7.5)	0	4 (5.8)	
LDH (%)					0.000
Not elevated	81 (46.3)	37 (55.2)	26 (66.7)	18 (26.1)	
Elevated (250–500)	34 (19.4)	12 (17.9)	9 (23.1)	13 (18.8)	
Elevated (>500)	51 (29.1)	17 (25.4)	1 (2.6)	33 (47.8)	
Unknown	9 (5.1)	1 (1.5)	3 (7.7)	5 (7.2)	

* A rank-sum test for the median time from diagnosis to stage IV disease was used to test the difference between groups of patients. WHO performance score: World-Health Organization performance score. LDH: lactate dehydrogenase.

## Supplementary Materials

The following are available online at https://www.mdpi.com/2072-6694/11/7/1007/s1, Figure S1: Kaplan-Meier Estimates per treatment strategy and level of LDH (normal LDH < 250 U/L vs. elevated LDH > 250 U/L).
