# Supplementary material for: Metastatic Uveal Melanoma: Treatment Strategies and Survival—Results from the Dutch Melanoma Treatment Registry
Source: Cancers (Basel). 2019 Jul 18;11(7):1007. doi: 10.3390/cancers11071007 (PMC6678641; doi:10.3390/cancers11071007)
Supplement: Supplementary file 1 [file cancers-11-01007-s001.pdf]

Supplementary Materials: Metastatic Uveal Melanoma: Treatment Strategies and Survival. Results from the Dutch Melanoma Treatment Registry

Anouk Jochems, Monique K. van der Kooij, Marta Fiocco, Maartje G. Schouwenburg, Maureen J. Aarts, Alexander C.J. van Akkooi, Franchette W.P.J. van den Berkmortel, Christian U. Blank, Alfonsus J.M. van den Eertwegh, Margreet G. Franken, JanWillem B. de Groot, John B.A.G. Haanen, Geke A.P. Hospers, Rutger H. Koornstra, Wim H.J. Kruit, Marieke Louwman, Djura Piersma, Rozemarijn S. van Rijn, Karijn P.M. Suijkerbuijk, Albert J. ten Tije, Gerard Vreugdehil, Michel W.J.M. Wouters, Michiel C.T. van Zeijl, Koos van der Hoeven and Ellen Kapiteijn

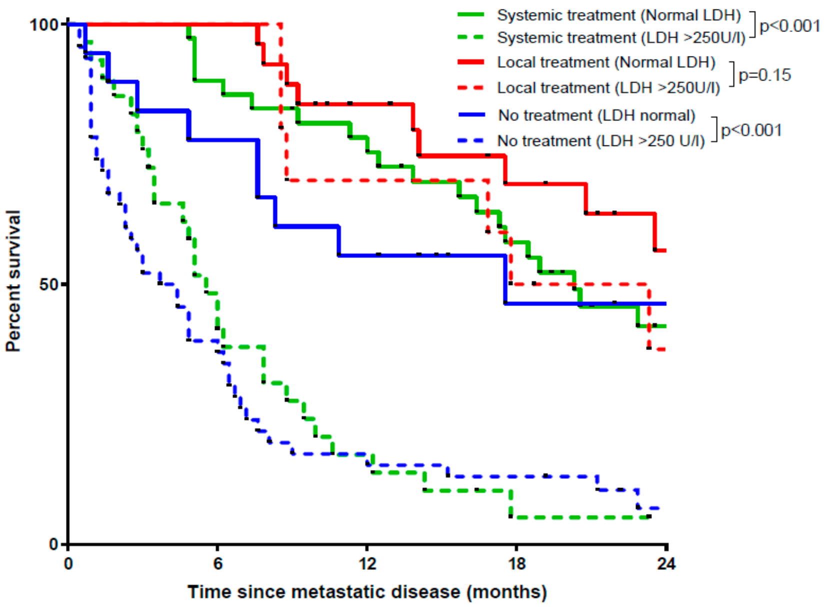

Figure S1. Kaplan-Meier Estimates per treatment strategy and level of LDH (normal LDH < 250 U/L vs. elevated LDH > 250 U/L).

© 2019 by the authors. Licensee MDPI, Basel, Switzerland. This article is an open access article distributed under the terms and conditions of the Creative Commons Attribution (CC BY) license (http://creativecommons.org/licenses/by/4.0/).
